# Splitting Mealtime Insulin Doses for Mixed Fat and Protein Meals in Children and Adolescents with Type 1 Diabetes Using Multiple Daily Injection Regimen: A Randomized Cross-Over Trial

**DOI:** 10.1155/2023/7467652

**Published:** 2023-10-27

**Authors:** Ahmed M. Hegab, Susana E. Hasaballah, Montaser M. Mohamed

**Affiliations:** Pediatrics Department, Faculty of Medicine, Sohag University, Sohag, Egypt

## Abstract

**Aims:**

Assessment of the glycemic outcomes of increasing and splitting mealtime insulin doses for mixed fat and protein meals in pediatric patients with type 1 diabetes mellitus (T1DM) using multiple daily injection regimen and comparing the effects of regular insulin and fast-acting insulin on glycemic outcomes following those meals.

**Methods:**

This single-center, randomized, cross-over trial included 43 children and adolescents with T1DM randomly assigned to receive three interventional insulin doses for lunch meals over 3 consecutive days; Intervention A (100% insulin-to-carbohydrate ratio (ICR) dose given as premeal insulin lispro with an additional insulin sensitivity factor-calculated correction dose after 3 hr), Intervention B (130% ICR dose split into 60% premeal insulin lispro and 40% postmeal insulin lispro after 30 min), and Intervention C (130% ICR dose split into 60% premeal insulin lispro and 40% postmeal regular insulin after 30 min). The test meal consisted of two slices of pizza (weight: 150 g, carbohydrates: 40 g, fat: 15 g, protein: 20 g, and calories: 380 kcal). Postprandial blood glucose levels were monitored for 6 hr.

**Results:**

There were no significant differences in postprandial blood glucose excursions following the three interventions. However, Intervention C had a significantly lower late (3–6 hr) blood glucose area under the curve (*p*=0.01). Postprandial hypoglycemia developed in 12 participants (27.9%) following Interventions A and B and in 17 participants (39.5%) following Intervention C (*p*=0.32).

**Conclusions:**

Using regular insulin as a postmeal portion of increased and split insulin doses provided better late postprandial glycemic outcomes following mixed fat and protein meals. However, the amount of additional insulin used needs optimization to reduce the frequency of postprandial hypoglycemia. This trial is registered with NCT04783376.

## 1. Introduction

The current management of type 1 diabetes mellitus (T1DM) involves the use of intensive insulin therapy—either by insulin pump therapy or multiple daily injection (MDI) therapy—and carbohydrate counting [1]. The mealtime bolus insulin doses are determined according to the amount of carbohydrates in each meal or snack. Insulin doses are calculated by dividing the amount of carbohydrates in grams by the insulin-to-carbohydrate ratio (ICR), which is the amount of carbohydrates in grams that can be covered by one unit of insulin [2].

Although carbohydrates are the main macronutrient that affects blood glucose levels, several studies demonstrated that dietary fat and protein can also affect postprandial glycemic responses [3]. It was found that high-fat meals usually result in delayed gastric emptying shortly after meals and induce an increase in insulin resistance in the late postprandial period [4, 5]. Protein usually does not affect the blood glucose level if consumed in small amounts [6]. However, high-protein meals can increase postprandial blood glucose levels [7]. The effect of fat and protein is additive with higher blood glucose excursions with increased fat and protein contents of meals [8].

Several studies suggested that more insulin was required to cover the fat and protein contents of meals [3, 7]. However, there is still a controversy about the appropriate amount of insulin that has to be added [9]. Pankowska and Blazik proposed an equation for calculating the insulin dose required to cover dietary fat and protein by which the additional insulin was calculated as the fat–protein units (FPUs) of the meal (each FPU contains 100 kcal of fat and/or protein) multiplied by the insulin ratio defined as the units of insulin that cover 10 g of carbohydrate [10]. Other authors suggested the addition of about 15%–40% of the ICR-calculated dose to cover the fat and protein contents of meals [11–14].

However, increasing the insulin dose alone is not enough to provide optimum glycemic control following fat and protein meals. The timing of insulin dose can also affect the postprandial blood glucose levels [12]. The increased insulin dose given before high-fat and protein meals could result in early postprandial hypoglycemia as the delayed gastric emptying induced by dietary fat produces a lag in glucose absorption [15]. Furthermore, blood glucose levels may remain elevated for several hours after high-fat and protein meals [16].

Only a few previous studies were conducted to evaluate different insulin dosing strategies for high-fat and protein meals in children and adolescents with T1DM using the MDI regimen [17–20]. Therefore, this study aimed to assess the glycemic outcomes of increasing and splitting mealtime insulin doses for mixed fat and protein meals in children and adolescents with T1DM using the MDI regimen. The study also aimed to compare the glycemic outcomes of using regular insulin and fast-acting insulin as the postmeal portions of the split insulin doses for mixed fat and protein meals.

## 2. Patients and Methods

### 2.1. Study Design and Settings

This was a single-center, randomized, cross-over, open-label, clinical trial conducted over 6 months from April 2021 to October 2021 at the Pediatrics Department, Sohag University Hospital, Sohag, Egypt.

### 2.2. Study Population

Children and adolescents aged 6–18 years, diagnosed with T1DM for at least 1 year, attending the pediatric diabetes clinic at Sohag University Hospital and using the MDI regimen with carbohydrate counting for at least 6 months were eligible. Children and adolescents with associated diseases such as autoimmune hypothyroidism or celiac disease and those with diabetes-related complications such as diabetic neuropathy, retinopathy, or nephropathy were excluded.

### 2.3. Ethical Considerations

The study protocol was approved by the medical research ethics committee at Sohag Faculty of Medicine (Institutional Review Board approval number: Soh-Med-21-01-02). The study was performed in line with the principles of the Declaration of Helsinki. Written informed consent was obtained from the parents or the legal guardians of each study participant. The study was registered at ClinicalTrials.gov (No. NCT04783376).

### 2.4. Assessment of the Study Participants

The participants were admitted to the Pediatrics Department at Sohag University Hospital for 1 week. Full history taking, thorough clinical examination, and review of the participants' medical files were done.

Adjustments of insulin doses, ICR, and insulin sensitivity factor (ISF) were done if required. The basal insulin dose was considered as accurate if the participant had fasting morning blood glucose levels between 70 and 130 mg/dL without overnight hypoglycemia [21]. ICR was considered as accurate if the blood glucose levels remained within 30 mg/dL of the premeal level 3 hr after the bolus dose [2]. ISF was considered as accurate if the blood glucose levels returned to the target range 3 hr after the correction doses [22].

### 2.5. Study Procedure

#### 2.5.1. Randomization

Each participant received three interventional insulin doses for the test meals in a random sequence by the withdrawal of a sealed envelope containing a paper with a written random sequence for the three study interventions generated by computer software. The envelope was withdrawn randomly and opened immediately before the first intervention and the sequence of interventions was followed as written.

#### 2.5.2. The Test Meal

The meal consisted of two slices of pizza topped with tomato sauce, mozzarella cheese, and minced beef (weight: 150 g, carbohydrates: 40 g, fat: 15 g, protein: 20 g, and total calories: 380 kcal). The meal was consumed within 20 min. Three lunch meals on three consecutive days were consumed by each participant at noon.

#### 2.5.3. Study Interventions

Each participant was assigned to receive the following three interventional mealtime insulin doses in a random sequence over the three study days:

*(1) Intervention A*. A 100% ICR dose given 10 min before the meal as fast-acting insulin (insulin lispro). If hyperglycemia (blood glucose level > 180 mg/dL) developed 3 hr after the test meal, an additional correction dose of insulin lispro was given according to the ISF.

*(2) Intervention B*. A 130% ICR dose split into a 60% premeal portion given 10 min before the meal and a 40% postmeal portion given 30 min after the premeal dose. Both portions were given as fast-acting insulin (insulin lispro).

*(3) Intervention C*. A 130% ICR dose split similar to Intervention B with the use of regular insulin as the postmeal portion of the dose.

#### 2.5.4. Insulin Administration

The types of insulin used in the study were the fast-acting insulin analog: insulin lispro (Humalog 100 units/mL) and regular insulin (Humulin R 100 units/mL). The insulin dose was given using half-unit increments insulin pens. Premeal bolus doses were injected subcutaneously into the abdomen, whereas the postmeal doses were injected into the upper quadrants of the buttocks. Injection sites were checked before injections with the avoidance of injecting insulin into lumpy or hypertrophied areas. All of the study participants used insulin degludec 100 units/mL (Tresiba) as a once-daily basal dose at night (8–10 PM). Correction insulin doses (insulin lispro) were given 3 hr before the test meals if needed to bring the blood glucose levels to the target range (70–180 mg/dL) before the test meals.

#### 2.5.5. Monitoring of the Study Participants

Following the meal consumption, participants were allowed to perform only sedentary activities. No food or drink— except water—was permitted for the following 6 hr unless needed to correct hypoglycemia. Capillary blood glucose levels were measured by a calibrated finger-prick blood glucose meter before the meal and then hourly for the next 6 hr after the test meals.

#### 2.5.6. Outcome Measures

The blood glucose excursion throughout the 6-hr postprandial period was the primary outcome measure of the study.

Secondary outcome measures included the time to peak and time to lowest blood glucose levels, blood glucose area under the curve (AUC), and the frequency of postprandial hypoglycemia among the study participants. The capillary blood glucose AUC was calculated using the linear trapezoidal method [23].

#### 2.5.7. Safety Outcome Measures and Management of Adverse Events

Mild hypoglycemia was defined as a blood glucose level of <70 mg/dL associated with any symptoms of hypoglycemia other than disturbed consciousness. Severe hypoglycemia was defined as a blood glucose level of <70 mg/dL associated with disturbed consciousness and required the help of others [24].

The study participants were under observation in the pediatric department wards throughout the follow-up period. At least one of the pediatric diabetes management team members at our department attended throughout the test to ensure the participant's compliance with the test instructions and to manage any adverse events. Before the consumption of each test meal, the intervention was explained to the participants and their families and they were asked to follow the test instructions throughout the follow-up period. They were also informed about the symptoms of hypoglycemia and were asked to inform the attending physicians and nurses immediately about the development of any symptoms of hypoglycemia. The blood glucose measurements, any adverse events, and the participants' adherence to the test instructions were recorded by trained nurses.

Postprandial hypoglycemia was managed according to the International Society for Pediatric and Adolescents Diabetes clinical guideline for the management of hypoglycemia [24]. Mild hypoglycemia was treated with 15 g of oral glucose given as a sweetened fluid. Severe hypoglycemia associated with disturbed consciousness was treated with 2 mL/kg of intravenous dextrose 10%. Blood glucose level was measured 15 min after correction of hypoglycemia, and treatment was repeated if the blood glucose level persisted below 70 mg/dL.

### 2.6. Statistical Analysis

A sample size of 42 was considered as the number required to provide 90% power at the 5% significance level, to detect a potential difference in mean blood glucose levels of 36 mg/dL between each two interventions, assuming a within-subject standard deviation of differences in glucose levels of 50 mg/dL and a two-tail *t*-test.

Statistical analysis was done using IBM SPSS Statistics for Windows, version 22.0 (IBM Corp., Armonk, NY, USA). The analysis was conducted according to the intention-to-treat principle.

Data were presented as means ± standard deviations for continuous variables with normal distribution and as medians (interquartile ranges (IQRs)) for variables with nonparametric distributions. The Kolmogorov–Smirnov test was used to assess the normality of distributions of continuous variables. Categorical variables were expressed as numbers and percentages.

For comparisons between each two interventions, the paired sample *t*-test was used for continuous variables with normal distributions and the Wilcoxon signed-rank test was used for continuous variables with nonparametric distributions. The McNemar test was used to compare categorical variables between each two interventions. To compare the three interventions together, repeated measures analysis of the variance (ANOVA) was used for continuous variables with normal distributions and the Freidman test was used for continuous variables with nonparametric distributions. The Cochran *Q* test was used for comparing the categorical variables between the three interventions. *P* − value < 0.05 was set as statistically significant.

## 3. Results

**Figure 1:**
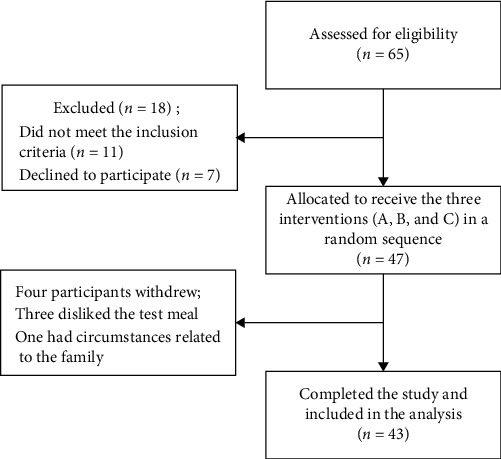
Flowchart of the study participants.

Forty-seven children and adolescents with T1DM participated in the study. Of whom, four participants withdrew; three did not like the test meal and one had circumstances related to the family. The remaining 43 participants completed the study and were included in the analysis. The flowchart of the study participants is shown in Figure 1.

Table 1 shows the clinical characteristics of the study participants. About 60% of the study participants were females. The median age (IQR) of the study participants was 12 (10–13) years, and the median duration of diabetes (IQR) was 3 (2–5) years. The ICR, ISF, total and basal daily insulin doses, and the HbA1c levels for the study participants are shown in Table 1.

### 3.1. Postprandial Glycemic Responses

Table 2 shows comparisons between the three insulin dosing interventions used with the study participants regarding the mealtime insulin doses and the postprandial glycemic responses. Intervention A had a significantly higher premeal dose and significantly lower postmeal and total insulin doses compared to the other two interventions (*p* < 0.001, for all). There were no significant differences in the premeal blood glucose levels between the three interventions (*p*=0.22).

Intervention C had significantly lower minimum blood glucose levels throughout the postprandial period compared to the other interventions (*p*=0.02). However, there were no significant differences between the three interventions regarding the time to the peak or the lowest blood glucose levels or the maximum blood glucose levels.

**Figure 2:**
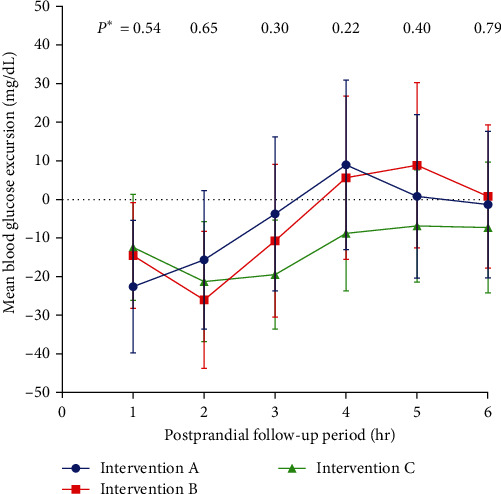
Postprandial blood glucose excursions following the three insulin dosing interventions used for the mixed fat and protein test meals among the study participants. Data are presented as means with 95% confidence intervals. *P* ^*∗*^ values were calculated by repeated measures analysis of the variance (ANOVA) test.

The mean postprandial blood glucose excursions for each intervention at 1-hr intervals for 6 hr after the test meals are shown in Figure 2. The mean blood glucose excursions decreased below the baseline blood glucose levels for 3 hr after the test meals with the three insulin dosing interventions. Following Interventions A and B, the mean blood glucose excursions reached above the baseline levels for 4 hr after the meal. However, the mean blood glucose excursions remained below the baseline blood glucose levels throughout the 6-hr follow-up period with Intervention C. There were no significant differences between the three interventions regarding the blood glucose excursions throughout the follow-up period.

### 3.2. Postprandial Blood Glucose Area under the Curve

The postprandial blood glucose AUC following the three insulin dosing interventions is shown in Table 3. Intervention C had significantly lower total and late blood glucose AUC compared to Intervention A (*p*=0.01 and 0.008, respectively) and a significantly lower late blood glucose AUC compared to Intervention B (*p*=0.02). There were no significant differences between Interventions A and B regarding early, late, or total blood glucose AUC.

### 3.3. Postprandial Hypoglycemia

Table 4 shows comparisons between the three insulin dosing interventions regarding postprandial hypoglycemia. Twelve participants (27.9%) had hypoglycemia with blood glucose levels of <70 mg/dL in the 6-hr follow-up period after Interventions A and B. Postprandial hypoglycemia with blood glucose levels of <70 mg/dL occurred in 17 participants (39.5%) after Intervention C. However, postprandial hypoglycemia with blood glucose levels below 54 mg/dL developed in three (7.0%) participants, two participants (4.7%), and four participants (9.3%) with Interventions A, B, and C, respectively. All of the detected episodes of postprandial hypoglycemia were corrected by oral glucose given as a sweetened fluid. None of the study participants required intravenous dextrose 10% for correction of hypoglycemia.

There were no significant differences between the three interventions regarding the percentage of participants with overall hypoglycemia or hypoglycemia with postprandial blood glucose levels of <54 mg/dL.

Eight out of 12 participants (66.6%) developed hypoglycemia with blood glucose levels of <70 mg/dL in the first 2 hr after the test meal with Intervention A. Moreover, 75% and 58.8% of participants with hypoglycemia had blood glucose levels below 70 mg/dL in the first 2 hr after the test meal with Interventions B and C, respectively. However, there were no significant differences between the three interventions regarding the time of development of postprandial hypoglycemia (*p*=0.84).

## 4. Discussion

The current study compared the effect of three different insulin dosing strategies on the postprandial glycemic responses following mixed fat and protein meals in children and adolescents with T1DM using the MDI regimen.

The study found that the mean blood glucose excursions decreased below the baseline blood glucose levels for the first 3 hr after the test meals with the three insulin dosing interventions whether the prandial insulin doses were given as 100% ICR or 130% ICR. These findings could be attributed to the delayed gastric emptying effect induced by dietary fat. Delayed gastric emptying might result in a lag in glucose absorption with an initial reduction in postprandial blood glucose levels followed by an increase in blood glucose excursions that extends for several hours after the meal [25, 26].

The current study found that the use of regular insulin as a postmeal portion of an increased (130% ICR) and split (60% premeal and 40% postmeal portions) insulin dose with mixed fat and protein meals provided lower late (3–6 hr) blood glucose AUC compared to a similarly increased and split dose using fast-acting insulin as pre- and postmeal portions as well as to a 100% ICR dose given entirely before the meal.

The current study was the first study to compare the effect of using regular insulin against fast-acting insulin analog as the postmeal portion of an increased insulin dose for mixed fat and protein meals in children and adolescents with T1DM using the MDI regimen. Regular insulin has a slower onset and longer duration of action [1]. Its use as a premeal insulin might result in higher blood glucose levels in the early postprandial period. However, its use might be advantageous for the prevention of the delayed postprandial blood glucose rise following mixed fat and protein meals.

Previous studies that assessed the effects of using regular insulin as a premeal insulin for high-fat and protein meals failed to find any benefit for its use over fast-acting insulin analogs. Jabłońska et al. [17] compared the effect of using fast-acting insulin and regular insulin for high-fat meals with both given as premeal insulin calculated according to the ICR. They found no benefit of using regular insulin instead of fast-acting insulin to control the postprandial hyperglycemic response following high-fat and protein meals [17]. Moreover, Smith et al. [18] reported that there was no benefit of using an increased insulin dose calculated as 125% of the ICR given as premeal regular insulin to overcome the postprandial glycemic response of high-fat and protein meals.

The study found that the use of an increased (130% ICR) and split fast-acting insulin doses for mixed fat and protein meals had no advantage over the use of the standard 100% ICR premeal fast-acting insulin doses with additional correction doses 3 hr after the meal. There were no significant differences between the two interventions regarding the mean postprandial blood glucose excursions, the time-to-peak blood glucose levels, or the postprandial blood glucose AUC.

In line with these findings, Campbell et al. [27] reported that using an additional 30% insulin aspart dose 3 hr after a high-carbohydrate and high-fat meal was effective in controlling the late postprandial hyperglycemic effects of dietary fat. They also found that this approach was not associated with an increase in the frequency of postprandial hypoglycemia compared to using a single 130% ICR insulin aspart dose before the meal [27].

The action profiles of fast-acting insulin analogs can affect the postprandial glycemic control following high-fat and protein meals. The peak action of fast-acting insulin analogs usually occurs within 1 hr, and their duration can vary between 4 and 6 hr [1]. However, the postprandial lipemia reaches its peak after about 3–4 hr leading to an acute reduction in insulin sensitivity in the peripheral tissues and an increase in the hepatic glucose output that may last for several hours after the meal [4, 28]. Consequently, the timing of splitting the fast-acting insulin doses with high-fat and protein meals needs to be optimized to provide better coverage for the postprandial glycemic responses following these meals. Frohock et al. [20] compared the effect of giving additional fast-acting insulin doses for dietary fat and protein before the meal, after 1 hr, or after 2 hr in 27 children and adolescents with T1DM using the MDI regimen. However, they found no significant differences in the postprandial glycemic control following the three interventions [20]. Nevertheless, further studies are still required to determine the proper timing for splitting prandial fast-acting insulin doses for high-fat and protein meals in children and adolescents using the MDI regimen.

The current study found that postprandial hypoglycemia was frequent and developed in about 28% of the participants following Interventions A and B and in about 40% of the participants following Intervention C. Most of these hypoglycemic episodes were mild and developed in the first 2 hr after the meals. This relatively high frequency of postprandial hypoglycemia could be attributed to the delayed gastric emptying effect of dietary fat and the increased insulin doses used with the meals.

Several previous studies found that increasing the prandial insulin dose was required to control the postprandial hyperglycemic responses following high-fat and protein meals [3, 7, 26]. Kaya et al. [19] demonstrated that mealtime insulin dosing based on carbohydrate plus fat and protein counting resulted in better postprandial glycemic control after high-fat and protein meals compared to insulin dosing based on carbohydrate counting only. Moreover, Smith et al. [14] reported that increasing the insulin doses for high-fat and protein meals resulted in a dose-dependent reduction in postprandial blood glucose levels and that using 140% ICR doses improved postprandial glycemic excursions without a significant increase in the frequency of postprandial hypoglycemia.

However, the use of additional insulin doses for high-fat and protein meals was associated with a higher frequency of postprandial hypoglycemia. Kordonouri et al. [15] demonstrated that using additional insulin to cover fat and protein contents of meals resulted in lower average blood glucose levels and significantly more frequent hypoglycemia compared to insulin doses calculated according to carbohydrate counting only. Similarly, Haak et al. [29] demonstrated that the use of additional prandial insulin for dietary fat and protein was associated with an increase in the percentage of hypoglycemia in adult patients with T1DM using insulin pumps. Moreover, Frohock et al. [20] reported that mild hypoglycemia developed in 55% of the study participants with the use of an additional 30% ICR insulin doses for high-fat and protein meals. On the other hand, Smith et al. [18] reported that using an increased insulin dose calculated as 125% of the ICR for high-fat and protein meals was safe in children and adolescents with T1DM using the MDI regimen without any increase in the risk of hypoglycemia whether the additional insulin was given before the meal or as an additional dose 1 hr after the meal. However, they found that the risk of hypoglycemia increased when this increased insulin dose was given as premeal regular insulin [18].

The findings of these studies suggest that the amount of additional insulin used for mixed fat and protein meals still needs to be optimized to avoid the risk of postprandial hypoglycemia. Moreover, the action profile of the prandial insulin used should also be considered in determining the amount and the timing for the additional insulin used with these meals.

The strength of the current study is that it was conducted in a hospital-based setting. This allowed accurate calculation of the insulin doses and better monitoring of the participants and minimized the effect of different external factors that might influence the postprandial blood glucose levels. This makes the results generalizable to a broader population of children and adolescents with T1DM using the MDI regimen.

However, the study had some limitations. First, the blood glucose levels were measured using finger-stick glucometers. The use of continuous glucose monitoring devices would have provided more details about the postprandial glycemic responses. Second, the study participants were monitored for 6 hr only after the test meals. A longer duration of postprandial blood glucose monitoring might have been required to assess the late glycemic response of mixed fat and protein meals.

## 5. Conclusion

In children and adolescents with T1DM using the MDI regimen, the use of regular insulin as postmeal portion of an increased (130% ICR) and split insulin dose provided lower late (3–6 hr) blood glucose AUC following mixed fat and protein meals compared to a similarly increased and split dose using fast-acting insulin as pre- and postmeal portions as well as to a 100% ICR dose given entirely before the meal. However, the amount of additional insulin to be used with these meals still needs to be optimized to reduce the frequency of postprandial hypoglycemia.

## Figures and Tables

**Table 1:** Clinical characteristics of the study participants.

Variables	
Gender Male, *n* (%) Female, *n* (%)	17 (39.5) 26 (60.5)
Age (year), median (IQR)	12 (10–13)
Duration of diabetes (years), median (IQR)	3 (2–5)
BMI (kg/m^2^), median (IQR)	16.7 (15–18.5)
Insulin-to-carbohydrate ratio (g/unit), median (IQR)	10 (7.5–15)
Insulin sensitivity factor (mg/dL per unit), median (IQR)	50 (45–80)
Total daily insulin dose (U/kg/day), mean ± SD	0.95 ± 0.09
Basal insulin dose (U/kg/day), median (IQR)	0.46 (0.43–0.49)
Basal dose to total daily insulin dose percentage (%), mean ± SD	47.6 ± 2.6
HbA1c level mmol/mol, median (IQR) %, median (IQR)	61.7 (58.5–66.1) 7.8 (7.5–8.2)

BMI, body mass index; hb A1c, glycated hemoglobin; IQR, interquartile range; SD, standard deviation.

**Table 2:** Comparisons between the three insulin dosing interventions used for the mixed fat and protein test meals regarding prandial insulin doses and postprandial glycemic responses among the study participants.

	Intervention A (100% ICR (lispro) ± 3-hr ISF-calculated correction dose (lispro)) (*n* = 43)	Intervention B (130% ICR; 60% premeal (lispro) + 40% postmeal (lispro)) (*n* = 43)	Intervention C (130% ICR; 60% premeal (lispro) + 40% postmeal (regular insulin)) (*n* = 43)	P1 value	P2 value	P3 value	P4 value
Premeal dose (units), median (IQR)	4.0 (2.5–5.5)	3.5 (2.0–4.5)	3.5 (2–4.5)	<0.001	<0.001	>0.99	<0.001
Postmeal dose (units), median (IQR)	0 (0–1.5)	2.0 (1.5–2.5)	2 (1.5–2.5)	<0.001	<0.001	>0.99	<0.001
Total dose (units), median (IQR)	4.0 (3.5–5.5)	5.5 (3.5–7.0)	5.5 (3.5–7.0)	<0.001	<0.001	>0.99	<0.001
Premeal BG level (mg/dL), median (IQR)	159 (125–175)	148 (117–174)	140 (120–165)	0.57	0.09	0.41	0.22
Peak postprandial BG level (mg/dL), median (IQR)	194 (150–230)	175 (124–208)	175 (130–218)	0.09	0.07	0.89	0.21
Time-to-peak postprandial BG level (hr), median (IQR)	4 (3–5)	4 (2–5)	4 (2–5)	0.66	0.06	0.31	0.34
Lowest postprandial BG level (mg/dL), median (IQR)	88 (68–115)	90 (65–122)	79 (63–106)	0.29	0.10	0.01	0.02
Time to lowest postprandial BG level (hr), median (IQR)	2 (1–4)	2 (1–3)	3 (2–5)	0.75	0.11	0.07	0.19
Participants with postprandial BG levels above 180 mg/dL, *n* (%)	27 (62.8%)	20 (46.5%)	21 (48.8%)	0.16	0.18	>0.99	0.13

BG, blood glucose; ICR, insulin-to-carbohydrate ratio, ISF, insulin sensitivity factor; IQR, interquartile range. P1: *p*-value for comparisons between Interventions A and B. P2: *p*-value for comparisons between Interventions A and C. P3: *p*-value for comparisons between Interventions B and C. P4: *p*-value for comparisons between the three interventions together.

**Table 3:** Comparisons between the three insulin dosing interventions used for the mixed fat and protein test meals regarding postprandial blood glucose area under the curve among the study participants.

	Intervention A (100% ICR (lispro) ± 3-hr ISF-calculated correction dose (lispro)) (*n* = 43)	Intervention B (130% ICR; 60% premeal (lispro) + 40% postmeal (lispro)) (*n* = 43)	Intervention C (130% ICR; 60% premeal (lispro) + 40% postmeal (regular insulin)) (*n* = 43)	P1 value	P2 value	P3 value	P4 value
Total (0–6 hr) blood glucose AUC (mg × hr/dL), mean ± SD	867.91 ± 230.74	842.24 ± 261.92	774.29 ± 200.21	0.54	0.01	0.07	0.04
Early (0–3 hr) blood glucose AUC (mg × hr/dL), mean ± SD	410.84 ± 100.41	393.63 ± 113.97	379.98 ± 114.57	0.31	0.15	0.47	0.35
Late (3–6 hr) blood glucose AUC (mg × hr/dL), mean ± SD	457.43 ± 160.80	448.62 ± 171.31	394.30 ± 123.07	0.76	0.008	0.02	0.01

AUC, area under the curve, ICR, insulin-to-carbohydrate ratio; ISF, insulin sensitivity factor; SD, standard deviation. P1: *p*-value for comparisons between Interventions A and B. P2: *p*-value for comparisons between Interventions A and C. P3: *p*-value for comparisons between Interventions B and C. P4: *p*-value for comparisons between the three interventions together.

**Table 4:** Comparisons between the three insulin dosing interventions used for the mixed fat and protein test meals regarding postprandial hypoglycemia among the study participants.

	Intervention A (100% ICR (lispro) ± 3-hr ISF-calculated correction dose (lispro)) (*n* = 43)	Intervention B (130% ICR; 60% premeal (lispro) + 40% postmeal (lispro)) (*n* = 43)	Intervention C (130% ICR; 60% premeal (lispro) + 40% postmeal (regular insulin)) (*n* = 43)	P1 value	P2 value	P3 value	P4 value
Participants with overall postprandial hypoglycemia (BG levels < 70 mg/dL), *n* (%)	12 (27.9%)	12 (27.9%)	17 (39.5%)	>0.99	0.33	0.26	0.32
Participants with early postprandial hypoglycemia (BG levels < 70 mg/dL in the first 2 hr after the meal), *n* (%)	8 (18.6%)	9 (20.9%)	10 (23.3%)	>0.99	0.77	>0.99	0.84
Participants with postprandial BG levels < 54 mg/dL, *n* (%)	3 (7.0%)	2 (4.7%)	4 (9.3%)	>0.99	>0.99	0.62	0.61

BG, blood glucose; ICR, insulin-to-carbohydrate ratio; ISF, insulin sensitivity factor. P1: *p*-value for comparisons between Interventions A and B. P2: *p*-value for comparisons between Interventions A and C. P3: *p*-value for comparisons between Interventions B and C. P4: *p*-value for comparisons between the three interventions together.

## Data Availability

The data that support the findings of this study are available from the corresponding author upon request.
